# The Session Wants and Need Outcome Measure: The Development of a Brief Outcome Measure for Single-Sessions of Web-Based Support

**DOI:** 10.3389/fpsyg.2021.748145

**Published:** 2021-10-29

**Authors:** Santiago de Ossorno Garcia, Louisa Salhi, Aaron Sefi, Terry Hanley

**Affiliations:** ^1^Kooth plc, London, United Kingdom; ^2^School of Psychology, University of Kent, Canterbury, United Kingdom; ^3^Department of Psychology, University of Exeter, Devon, United Kingdom; ^4^School of Environment, Education and Development, The University of Manchester, Manchester, United Kingdom

**Keywords:** single-session counselling, brief interventions, therapy outcome measure, iterative design, web-based therapy, digital mental health, patient-reported outcome, idiographic measurement

## Abstract

Single-session, brief interventions in therapy for young people make up a large proportion of service provision, including in digital mental health settings. Current nomothetic mental health measures are not specifically designed to capture the benefit or ‘change’ directly related to these brief interventions. As a consequence, we set out to design an outcome measure to concretely demonstrate the value of single-session interventions. The Session Wants and Needs Outcome Measure (SWAN-OM) aims to capture in-session goals and focuses on being user-centric, elements critical to the success of single-session and brief interventions which typically are asset-based and solution-focused. We describe the 4-stage process that was followed to develop this measure: (I) classical item generation and development, (II) content and (III) face validity pilot testing, and (IV) a user-experience approach with young people using framework analysis. This final stage was critical to ensure the integration of this outcome tool into a web-based digital therapy setting, a context which adds another layer of design complexity to item and measure development. This iterative methodology was used to overcome the challenges encountered and to place the needs of the young people and service practitioners at the centre of the design process, thus ensuring measure usability. To end, we highlight the main lessons learnt from engaging in this design process. Specifically, the needs of a measure for single-session interventions are considered, before outlining the learning associated with integrating the measure into a digital mental health platform. Both of these areas are emerging fields and, as such, this study contributes to our understanding of how an idiographic patient outcome theory driven measure can be created for use in a web-based digital mental health therapy service.

## Introduction

For many who seek mental health support, one session with a therapist can prove sufficient (Hymmen et al., 2013), with 58.6% of individuals in one study reporting the single-session met their needs after a 12-month follow-up period (Hoyt et al., 1992). Therefore, as many service users will engage in potentially only one session, the impact of any therapeutic intervention cannot be measured or followed up on in the traditional way using pre-post measurement after a reasonable period since the intervention took place. Mental health services typically utilise a battery of nomothetic clinical measures to track patient needs, progress and intervention outcomes (Sales and Alves, 2016; Lloyd et al., 2019). These have been very useful in more traditional clinical settings which offer longer, structured interventions, such as those seen in the United Kingdom, in Child and Adolescent Mental Health Services (CAMHS) and within change programs, such as Improving Access to Psychological Therapies (CYP-IAPT) (Batty et al., 2013). However, even though the majority of sessions is part of structured interventions in CAMHS, a large proportion of the children and young people population still only use the service for one single-session (Edbrooke-Childs et al., 2021). Therefore, alongside more nomothetic measures, patient-centred outcomes are being increasingly used (Collins, 2019). These routinely collected Patient-Reported Outcome Measures (PROMs) have been seen to be useful in mental health settings and provide the patient with a more holistic progress measure that reduces treatment failur and enhances positive effects of the intervention (Brattland et al., 2018; Lambert and Harmon, 2018). PROMs are important as they align more with patient-perceived change and progress rather than measurement being centred around the clinician perceived symptomatic progress (Collins, 2019; Flannery and Jacob, 2020). Therefore, the use of nomothetic PROMs aims to give the patient a voice in their mental health journey, but they fail to capture individualised wants and needs in treatment and measure the person’s experience of change, which may not be shown in clinical measure alone. Idiographic PROMs (I-PROMs) are aligned more closely to the patient-centred approach to therapy (Wheat et al., 2018) and show promise to be transferable to digital mental health services, as they can provide tailored cues for therapy to each user, they appear to enable the person to ‘tell their story’, and are proven to improve patient-clinician communication (Greenhalgh et al., 2018). Therefore, in this paper, we discuss the development of an I-PROM for single-session therapeutic interventions in a digital mental health service.

### Digital Mental Health Access

Digital mental health settings are increasingly being accessed, partially due to the increased need and resourcing of mental health services (Mohr et al., 2013), but also perhaps more importantly due to the increased choice, offering and flexibility that digital mental health services present (Sweeney et al., 2019). Nomothetic PROMs are not always tested or designed with the digital mental health provision in mind, and they are often reported to be alienating by children and young people (Sharples et al., 2017). Furthermore, in an online therapeutic context, service users have been shown to score higher overall on nomothetic measures than the clinical population (Mindel et al., 2021).

Within web-based therapy services, similar to what is observed in CAMHS, a large proportion of the requests from service users is for single-session therapeutic support or drop-in therapeutic engagement with practitioners at the point of seeking support (Hanley et al., 2020). This type of therapeutic engagement requires an understanding of the wants and needs of the service user at that specific time, with a focus on collaborative problem solving (Schleider and Weisz, 2017; Fleming et al., 2018; Schleider et al., 2020a,b). Single-sessions and drop-in therapy sessions can be one-off or very intermittent and differ from a structured or ongoing therapeutic engagement that resembles traditional online web-based therapy or counselling programmes with regular sessions across time with a consistent practitioner (Chorpita, 2019).

### Measuring Single-Session Outcomes

Currently, there are known helpful aspects of single-session or drop-in therapeutic interventions. For example, a review by Hymmen et al. (2013) identified some of these as having the opportunity to talk about a problem, receiving helpful advice, feeling supported, being referred to other resources and having direct access to the service. However, even though there are known service user wants and preferences, there is not a sufficient outcome measurement for this type of intervention which translates these patient wants into achievable outcomes. There are limitations in measuring the outcomes of single-sessions as they are often stand-alone therapy sessions with no follow-up engagement opportunities. Within these therapeutic interventions, patients often have a specific focus, a need or aim to achieve something or find solutions; therefore, practitioners provide support or information directly related to client needs, with the awareness that an individual may only need one session to attain what they need (Dryden, 2020; Kachor and Brothwell, 2020).

To our knowledge, there are currently no suitable or specifically design outcome measures to provide evidence that single-sessions are useful to the service user. Both in digital and in face-to-face settings, there is limited feedback on whether the service user’s session was useful, sufficient or even if the user decided to withdraw from seeking therapy after their first session. Therefore, developing a measure for this specific type of intervention will be useful and applicable in a range of therapeutic services.

The use of nomothetic clinical measures is not a suitable or effective measure of progress in this scenario, whereas a more idiographic assessment that measures the individual service user wants and needs enables the outcome measure to be tailored to this type of single-session which differs across service users (Haynes et al., 2009). This is a problem for all single-session intervention but can be an even larger problem for digital mental health platforms where users can be anonymous and ‘drop-in’ to the service intermittently. Therefore, there is an overarching need for a suitable measure for single-session or drop-in therapeutic interventions that applies both to digital and face-to-face mental health services.

Hymmen et al. (2013) identified 18 studies looking at the effectiveness of single-session therapies, yet they only found six of the studies used standardised outcomes and from those six studies the standardised outcomes selected were diverse, reiterating the need for a specifically design outcome measure for single-session therapy.

Some single-sessions and brief interventions are targeted to specific problems and common mental health difficulties like anxiety, depression or addictions (Coverley et al., 1995; McCambridge and Strang, 2004; Weersing et al., 2017), making use of symptom-specific outcome measures like the Beck Depression Inventory (BDI–II; Beck et al., 1996), Pediatric Anxiety Rating Scale (Research Units on Pediatric Psychopharmacology Anxiety Study Group, 2002) or the Severity of Dependence Scale (Gossop et al., 1995). Others take a more general approach and select outcome measures that look at overall wellbeing measuring general health and functioning for children and young people (Perkins, 2006; Perkins and Scarlett, 2008). In either case, these measures lack the immediacy of change that is required for short-term interventions and may dilute its capabilities to demonstrate emotional changes and positive outcomes. To our knowledge, only one standardised instrument, the Counselling Progress and Depth Rating Instrument (Bagraith et al., 2010; Chardon et al., 2011), was designed to report counsellor integrity for the session and it was administered in online environments, but this was designed to analyse transcripts (Dowling and Rickwood, 2013), which makes it difficult to use as a routinely collected outcome measure. Many studies in single-session interventions rely on using individualised measures like clinical interviews and counsellor feedback to monitor treatment outcomes, for instance, the use of goals (Feldman and Dreher, 2012) and structured assessments (Denner and Reeves, 1997). In their review, Beidas and colleagues (2015) identified different instruments used for treatment monitoring and evaluation, such as the Brief Problem Checklist (Chorpita et al., 2010), Pediatric Symptom Checklist/Youth Report (PSC & Y-PSC; Jellinek et al., 1988) or the Strength and Difficulties Questionnaire (Goodman, 2001). The Youth Counselling Impact Scale (YCIS; Riemer and Kearns, 2010) may be suitable for tracking outcomes and alliance for each session, but not extensively used in single-sessions, brief interventions or digital contexts. Such findings demonstrate the lack of consensus in outcome measure use for single-session interventions. Other studies have also used standardised questionnaires that focus on the experience of the service, such as the Client Satisfaction Questionnaire (Larsen et al., 1979), or general functioning and wellbeing scales, for example, the Outcome and Session Rating Scales (Duncan et al., 2003; Bringhurst et al., 2006), in an attempt to measure the effectiveness of single-sessions (Perkins and Scarlett, 2008; Kachor and Brothwell, 2020). Overall, the variety of methods that aim to capture meaningful change or outcomes from single-sessions demonstrates a lack of consistency in such measurement and highlights the need for a tailor-made outcome measure for this type of therapeutic intervention.

### The Framework for Creating a Single-Session Measure

To make a single-session outcome measure scalable, the measure needs to be rooted in the theoretical rationale for why children and young people attend single-sessions and what outcomes are achievable in a single-session. Examining common needs among the population who access single-sessions will allow aggregation of needs being met as an outcome and their wants for these sessions. Yet we aim to retain the user-centred approach to allow tailored responses from the individual alongside the common needs. This is important as these sessions are very individualised to the wants and needs of each service user (Schleider et al., 2020b).

The measure development of the session wants and needs outcome measure (SWAN-OM) was developed in conjunction with a web-based therapy service (Kooth which is a web-based digital mental health platform for Children and Young People). In 2019, Kooth undertook a Theory of Change study to examine the key ingredients of web-based support. This method was chosen to evaluate the service to reflect the non-pathologised nature of the service, to examine the wants and needs of users rather than clinical diagnoses, the evaluation described a high-outcome level matrix representing the common wants and needs often seen in the service. In consultation with practitioners, academics and service users, the study identified input, process and outcomes for distinct different elements of the service and interventions, one of which was focussed around ‘responsive support’, the delivery of drop-in, often single-sessions of support through chat (Hanley et al., 2020).

The Theory of Change thus helped identify some key indicators of change and impact in single-sessions, that reflected the wants and needs of users through the analysis of transcribed sessions of single-session support and drop-in, developing a framework of outcomes that are commonly seen in the service for these types of brief intervention.

From examining Kooth’s Theory of Change (Hanley et al., 2020; Hanley et al., 2021), it was apparent that an I-PROM measure will be a suitable measure option. This will provide an individualised tool that explores changes in ‘in-session’ goals around wants and needs. Overall this measurement tool aims to provide a marker for the service user about what they want to achieve from their session and encourages reflection after their session to determine if they achieved what they wanted, providing a solution-focused framework for the single-session. A relational, person-centred measure was needed that enabled reflection of session progress, in-session goals. The measure needed to be asset-based that related to the wants and needs of the service user rather than symptom focus. This measure, along with other potential tracking outcomes, aims to be a useful therapeutic tool, providing a which can be used as a cue for a therapist when is seen by the practitioners. The use of an I-PROM for single-sessions will also facilitate routinely data collection. So aggregated data from the measure across service users can be collected for monitoring data on the analysis of this I-PROM by aggregating the scores of the common goals that you can find in the instrument, while providing the option to develop individual goals (Jacob et al., 2021). I-PROMs have increased currency especially for person-centred support service provision within children and young people population, while other measures can be used, no idiographic measure exists or has been designed specifically for single-session work.

In developing the SWAN-OM measure, there were unique demands on this measurement tool which contributed to the outcome design and methodology given the digital setting. Some of these demands may seem unique to a web-based therapy service; however, increasingly outcome measurement tools used in face-to-face services also need to be user friendly on digital technology, providing acceptable and to use outcome tools on electronic devices. This poses an additional layer of complexity when designing a person-centred outcome measure to ensure it meets digital accessibility standards and can be used in therapeutic settings. Consequently, to overcome these challenges, a unique design process was undertaken. Due to the fast-paced nature of digital settings and the importance of user acceptability and engagement with this measure, an iterative, phased approach was favoured for the development of the instrument. This took an item generation approach based on a Theory of Change for web-based therapy service, then utilised content (Zamanzadeh et al., 2015) and face validity testing (Allen and Yen, 1979; Nevo, 1985; Anastasi and Urbina, 1997; Hardesty and Bearden, 2004), to finally conduct user-experience testing to design using framework analysis (Ritchie and Spencer, 1994; Ritchie and Lewis 2003), with the focus to integrate this outcome tool into the web-based therapy service. This type of methodology aims to accelerate the process from inception-to-design-to-realisation (Honary et al., 2018) and is useful when designing for digital settings where human-computer interactions are important to examine. The rationale behind this methodological choice is that there are three critical phases to the development process: create, trial and sustain. Within each of these, there are design and evaluation elements to iteratively improve the tool of measurement following best practices (Mohr et al., 2017; Boateng et al., 2018) and taking into consideration the applied context of a web-based service. For each of these phases, we involved different stakeholders to reflect and provide feedback on the design processes. This provided the views and perspective of service users and practitioners, both of whom have experience with single-session therapeutic interventions and would have valuable insight into the acceptability and effectiveness of the new SWAN-OM instrument. This design process aims to put emphasis on the user when designing mental health outcome measures (Honary et al., 2018) and their participation.

Therefore, in this paper, we showcase the design of a novel I-PROM, the SWAN-OM. There were specific considerations for application to web-based services by using a user-centred and participatory approach for its design. Single-session and drop-in interventions in mental health services are common among children and young people when accessing these services. There is a substantial gap in how to measure these types of interventions and not many instruments designed to overcome this gap. Digital web-based mental health service adoption is increasing across populations providing an accessible way for people to access emotional support and counselling online. The use of outcome measures is proven beneficial for counselling and therapeutic sessions in general, but none of those outcome measures has been designed for a digital context delivering single-session interventions. This manuscript describes the methodologies and iterative design that researchers conducted inside a digital web-based therapy service, linking user experience with classical questionnaire development theory to illustrate the creation of the SWAN-OM in four design phases. We discuss valuable lessons learnt from this novel design process and challenges that researchers may encounter when designing instruments for web-based therapy services and digital interventions more broadly.

## Methods and Findings

To design the SWAN-OM, a four-phase design process was conducted with different data collection methods pertinent to each phase: starting with (I) item creation using domains and construct definition to determine the items within the scale. Following the creation of the individual items and the item domains, these were then tested to examine (II) content validity. This involved experts to assess the ability of the items in measuring the properties of the measured constructs. From this stage (III), face validity was examined by consulting with both service users and practitioners through piloting the instrument within the service. Then finally, of high importance when developing a measure for a digital platform is (IV) user-experience testing. This was conducted through participatory workshops in conjunction with young people who represented digital mental health service users. At each phase of development, there are iterative changes and improvements to the measure as well as the enquiry to the different stakeholders that utilise the measure.

In each of the phases, the methods used are described sequentially in sections alongside the findings that supported the methodological design of each phase. This demonstrates the iterative decisions made during the measure development.

### Phase I: Item Generation

Items were generated to represent the main wants and needs of young people from single-sessions or drop-in sessions. Initially, four domains of wants and needs were identified from Kooth’s Theory of Change that represent the needs for mental health support in children and young people that access the web-based therapy service (Hanley et al., 2020; Hanley et al., 2021). Such domains were also previously explored investigating the goals collaboratively set by young people at the onset of their contact with the service (Hanley et al., 2017; Jacob et al., 2020). These domains allowed to set the parameters of the constructs that the measure will be seeking to capture concerning what children and young people seek in an online service, for example, whether individuals seek informational or emotional support and whether this support was directed to support interpersonal or intrapersonal change.

**Figure 1:**
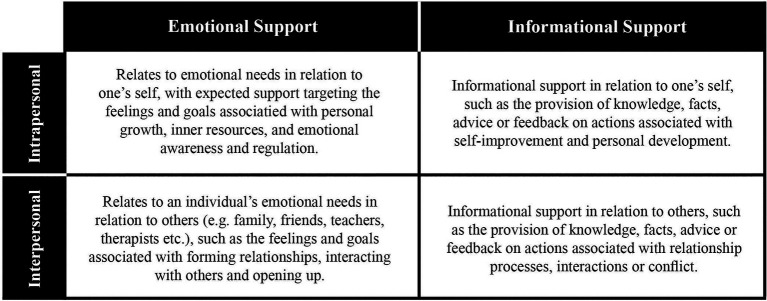
Kooth high-level outcome matrix – Domains for SWAN-OM.

**Figure 2:**
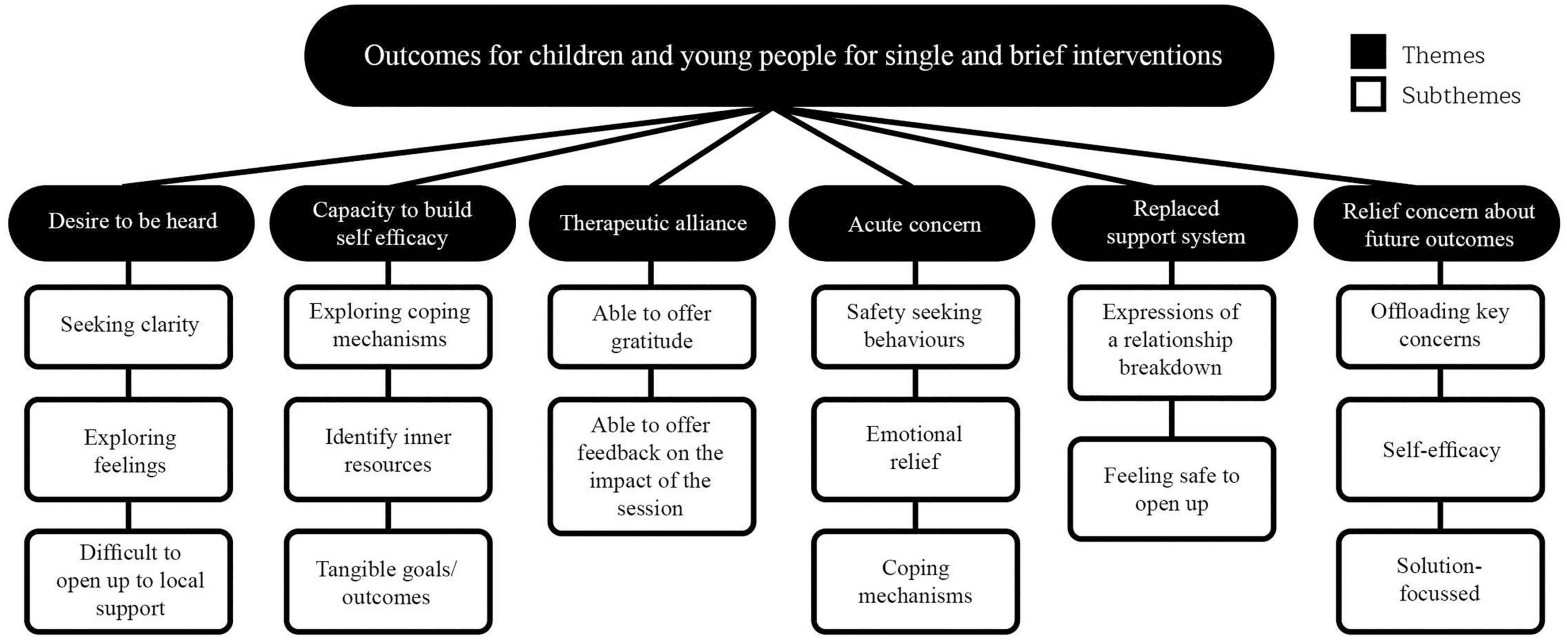
Thematic tree for single and drop-in brief interventions in Kooth.

These four domains (see Figure 1) were used alongside a framework which was derived from qualitative thematic exploration of transcripts from children and young people. Importantly for the SWAN-OM development, there was a focus on only service users who engaged with Kooth in a single-session or a brief manner over a non-specific period of time without accepting or being offered structured sessions of support (see Figure 2). Both the domains and the framework enabled the initial item generation. The main aims of these items within this measure were to help the young person to articulate what they wanted or needed from the session and capture if their in-session goals were met after that session.

#### Participants

Two researchers (female and male) from the Kooth research team took part in this phase. The two researchers knew each other and had worked on similar research projects previously. Both researchers had previous knowledge of Kooth as a service and reviewed the literature and data available on Theory of Change developed for Kooth.

#### Design and Procedure

Firstly, the two researchers independently coded the themes from the tree (Figure 2) in each domain of the outcome matrix (Figure 1), the coding was shared in structured meetings to achieve coherence between items, themes and domains.

The researchers reached consensus using the nominal group technique (Van de Ven and Delbecq, 1972) to resolve any discrepancies from their independent findings, raising issues and discussing the discrepancies systematically to achieve a list of coded items with the respective theme and domain from the matrix (see Supplementary Table S1). A combination of deductive and inductive approaches was applied for each domain and theme to generate items that reflected the theme and domain of interest. Despite some instruments with similar statements being identified through the literature review and assessment process, most of the statements were generated in an inductive manner using the transcript data generated in the thematic tree within the framework.

Each item was composed of two paired statements, the first is presented before a therapeutic session asking about the in-session goals (the service user’s want or need for that session). These correspond to the domain and theme and were prompted by the overarching question; ‘Why have you come to chat today?’. The second is a follow-up statement presented after the therapeutic session that aims to capture if the in-session goals (want or needs for that session) were achieved. This is captured on a 10-point Likert scale.

#### Results

A set of 46 item statements were generated for the domains and themes identified in both frameworks (See Supplementary Table S1). This set of statements was constructed into a pool of items used to be further validated in the next phase of development through content validity approaches and expert assessment.

### Phase II: Content Validity

Validity is defined as the instrument’s ability to measure the properties of the construct under study (DeVon et al., 2007). More specifically, content validity is the ability of the items to represent the domain intended to be measured (Zamanzadeh et al., 2015). Experts are often used to judge the content of the measure to determine the clarity or comprehensiveness of each item, as well as the relevance based on the domain and aim of the measure.

The aim of these workshops was to determine the extent to which the items were representative of the outcomes of single-sessions and brief interventions at Kooth defined by the four outcome domains representing the wants and needs commonly found in users seeking online mental health support within the service.

#### Participants

To assess content validity, a group of eight experts from the research, clinical and product teams at Kooth was recruited. The experts formed an expert reference group (ERG). Experts were required to have previous knowledge of Kooth’s Theory of Change (Hanley et al., 2020) and recognised experience within the service for at least 1year; all experts were part of the same organisation with a previous working relationship.

#### Design and Procedure

Item Content Validity indexes (CVI) were assessed through an online questionnaire and a workshop with ERG members, the survey was administered in advance to the workshop to all experts who provided their CVI scores on the initial pool of items proposed and the CVI findings between experts were discussed in a remote workshop using Zoom^1^ in order to make decision on exclusion, inclusion and modification of items after the ERG panel review.

##### Item Content Validity Questionnaire

The ERG completed an online questionnaire that asked them to rate each question on its relevance and clarity, the questionnaire contained the initial 46 items developed in phase 1 to be scored by the experts. Each question contained a 4-point Likert-scale response for both relevance and clarity (i.e. relevance responses on a scale from not relevant, somewhat relevant, quite relevant to highly relevant and clarity responses on a scale from not clear, somewhat clear, quite clear to highly clear). An open-ended text box was also provided for the experts to add comments or offer suggestions, or examples to rephrase an item.

##### Item Content Validity Index Calculations

The Item Content Validity Index (I-CVI) of each indicator for both relevance and clarity was calculated (calculated from the questionnaire responses). This involves calculating the sum of the experts scores for each item and dividing by the number of experts (Zamanzadeh et al., 2015). Items that scored equal to or over 0.75 on both relevance and clarity were automatically included for the next round. If both relevance or clarity received a score of lower than 0.5, the item was excluded, and if one or both items were between 0.5 and 0.75 the item was included in the list to review in the workshop.

The relevance scale-CVI/Ave (S-CVI/Ave), which is the average of the I-CVIs for all items on the scale, was also calculated for each high-level outcome matrix domain (Table 1). The average item quality is important to examine as we are interested in item quality, rather than the average performance given by the experts (Polit and Beck, 2006). The gold standard of acceptable CVI scores and the number of experts required to ascertain robust calculations has been hotly debated in the literature, with a recommended number of experts ranging between two and nine, and CVI between 0.78 and 1 for excellent content validity (Yusoff, 2019).

**Table 1:** I-CVI scores for each item selected from the expert workshops.

Item	Statement pre-chat	Statement post-chat	I-CVI Relevance	I-CVI Clarity
**Emotional interpersonal domain**				
Item 1	To feel safe to tell others in my life what is going on	I now feel safer to tell others what is going on	0.875	0.75
Item 2	To share my story with another person*	I felt comfortable sharing my story with another person*	0.625	0.75
Item 3	To feel comfortable opening up to people in my life	I feel more comfortable about the idea of opening up to people in my life	0.625	0.875
Item 4	To feel comfortable accessing support offline	I now feel more comfortable asking for support offline	1	1
Item 5	To work through some difficulties in my relationships*	I have started to work through some difficulties in my relationships*	0.75	0.375
Item 6	To be able to understand others	I was able to ask questions to help me to understand others	0.75	0.75
Item 7	To explore my problems with someone	I was able to tell someone about my problems	0.875	1
**Emotional intrapersonal domain**				
Item 8	A safe space to explore how I feel	I got a safe space to explore how I feel	1	0.875
Item 9	To feel better now	I feel better	0.875	0.75
Item 10	To discover how I can help myself to feel better	I can now help myself feel better	1	0.75
Item 11	To talk about something I have not told anyone before	I was able to talk about something I have not told anyone before	0.875	0.875
Item 12	To explore what is possible on Kooth	I understand what is possible on Kooth	0.875	0.875
Item 13	To be able to work out a situation I am in	I was able to work out the situation I was in	0.75	0.875
Item 14	To be ok with my feelings	I am ok with my feelings	0.75	0.75
Item 15	To feel listened to	I felt listened to	0.875	0.875
Item 16	To explore how I feel	I was able to open up about my feelings	0.875	1
Item 17	To feel in control of how I receive my support	I had a say in what we talked about	0.875	0.75
Item 18	To understand my feelings and behaviours*	I understand my feelings and behaviours better*	0.75	0.5
**Informational interpersonal domain**				
Item 20	Discover how to find the people who can help me	I can now identify people who might be able to help me	0.75	0.875
Item 21	To learn how to relate with others	I can identify new ways to relate to others	0.75	0.75
Item 22	To identify goals that will help me improve my relationships	I know the steps to take to improve my relationships	0.75	0.875
Item 23	To understand or improve my relationships with others	I have the tools to better understand my relationships with others	0.875	0.875
Item 24	To learn how to manage conflict with others	I now feel more confident managing conflict with others	1	1
Item 25	To identify solutions to manage my relationships*	I have identified possible solutions to manage my relationships*	0.875	0.625
Item 26	To find out how useful it is to talk to someone*	It was useful to talk to someone*	0.75	0.625
**Informational intrapersonal domain**				
Item 27	Some information about how to keep myself safe	I got some information about how to keep myself safe	0.875	0.875
Item 28	To find ways to help me worry less	I have found some ways to help me worry less	0.875	0.75
Item 29	To learn how to feel better	I have learned ways/skills to feel better	1	1
Item 30	Able to manage my situation better	I feel able to manage my situation better	1	0.75
Item 31	To identify ways I can help myself	I have identified ways to help myself	1	1
Item 32	To learn the steps to achieve something I want	I understand the steps to achieve my goal	0.875	0.875
Item 33	Information on how to feel more confident	I feel more confident in my abilities	0.75	0.75
Item 34	To identify a solution to a problem in my life*	I have found a possible solution to a problem in my life*	0.75	0.625

* *Items reviewed from the expert workshop I-CVI reported before inclusion*.

##### Expert Reference Group Workshops

An online workshop with the same expert panel (N=8) was then conducted to evaluate the items which attained a low-mid I-CVI score for either relevance or clarity. An important aspect of this workshop was to also investigate the qualitative feedback that experts provided through free-text around accuracy, interpretability and appropriateness. Each item for review was re-assessed by researchers and further proposals were created for each item to present in the workshop to the ERG using the qualitative information suggested in the questionnaire. The workshop presented each item indicator once at a time for its assessment, alongside revised versions, the workshop used a polling system to ask experts whether they: wanted to keep the original version, accept revisions proposed by the researchers or remove the item entirely.

#### Results

##### I-CVI Results

The initial results from the questionnaire showed a healthy range of content validity for each item, alongside highlighting the indicators where further revision was required or excluded. Nine items were excluded as they did not meet the criteria scoring below 0.75 for clarity or relevance in the I-CVI. Ten items were identified for review with the expert panel as their I-CVI ranged between 0.5 and 0.85 in clarity and relevance to determine its change, inclusion or exclusion from the measure. From this, 26 items above 0.75 in the I-CVI for clarity and relevance were included in the measure. A further calculation of S-CVI for each domain was also conducted presenting acceptable levels of content validity for each scale (Table 2).

**Table 2:** S-CVI/Ave scores for each quadrant.

	Emotional interpersonal	Emotional intrapersonal	Informational interpersonal	Informational intrapersonal
S-CVI	0.71	0.77	0.72	0.80

##### Expert Workshops Results

After the appropriate revisions based on the advice from the ERG workshop, the indicators were consolidated into a final list of 34 items to be included in the pilot study for the measure. I-CVI for each item relevance and clarity was calculated (Table 1). Twenty-seven items (79%) were marked as relevant and clear with I-CVI scores between 0.75 and 1 for both scales. Two items score below the threshold in relevance (I-CVI=0.625) but scored highly on clarity. In addition, five items scored below for clarity (I-CVI=0.5–0.625) but highly on relevance, these items were still included after the expert panel feedback. The majority of items scored in the instrument was considered clear and relevant; in addition to I-CVIs, Kappa scores were calculated for each item to control for agreement due to chance (See Supplementary Table S2). The results on the content validity indexes provided a good case to take the initial SWAN-OM to the next phase.

### Phase III: Face Validity

Face validity relates to how much responders judge the items to be an appropriate measure on the constructs that the scale intended to measure (Allen and Yen, 1979; Nevo, 1985; Anastasi and Urbina, 1997; Hardesty and Bearden, 2004). Therefore, in this phase, we now move from creating the measure to piloting the measure by examining the face validity of the instrument in the context it is intended to be used in. Here, we aim to examine the appropriateness of the measure for single-session and brief interventions. The measure was trialled in Kooth (a web-based therapy service) to examine the perspective of both service users (children and young people) and practitioners.

#### Pilot With Practitioners

##### Participants

Practitioners at Kooth volunteered to participate in the pilot study (N=7). The SWAN-OM was successfully administered to 89 distinct service users accessing Kooth service during six of piloting within the digital mental health service.

##### Design and Procedure

The selected 34-items developed in the content validity phase (phase 2) were trialled for 6weeks on the Kooth platform with eligible service users (piloted on new drop-in users, with the presumption that they would only use Kooth for a single-session or a series of brief interventions).

Two initial training sessions were conducted to familiarise the practitioners with the workflow for administering the measure during therapy sessions. Practitioners administered the measure manually in the web-based therapeutic chat sessions and recorded the change score and any feedback. Following acquiring consent, the practitioners presented the text ‘Why did you come to chat today?’ and presented the four initial questions, relating to each domain: (1) I want to explore more about how I relate to other people (emotional-interpersonal), (2) I want to understand myself more (emotional-intrapersonal), (3) I want to learn some skills to try with other people (informational-interpersonal) and (4) I want information about something important to me (informational-intrapersonal). When a young person selected a response, the practitioners selected the item indicators relating to the selected domain into the chat text box. These items aim to inform the practitioner of the service users wants and needs of that specific session.

As the chat and intervention neared its end, the practitioners provided the follow-up items that matched the young person’s initial selected items. Young people then rated how much they thought the chat had achieved the item aims, on a scale of 1–10. The practitioners also note down their observations about the measure, as well as any record of any feedback ascertained directly from the young person.

Two workshops were conducted during the pilot phase: one midway through to present initial findings and gain feedback, and one at the end to present overall findings and collate any further feedback. The workshop allowed reflections by participants that were used in future phases of the measure design.

#### Results

##### Pilot Test Results Within Kooth

From the 89 distinct service users who received the measure, there were a total of 196 administrations of the measure. From this, there were 164 ‘change scores’ recorded. There were 32 instances of no score recorded (N=15), in most cases because the user left the chat early or the connection dropped out before the follow-up was administered.

The pilot study revealed that service users were selecting indicators from the two intrapersonal domains over 91.8% of the time, with indicators in the interpersonal domains only selected a total of 16 times (Table 3). Over half of the participants involved in the pilot (55.6%) only selected one indicator (*n*=50), and 33.3% of participants selected between 2 and 4 indicators (two indicators: *n*=11; three indicators: *n*=10; and four indicators: *n*=9). Because there was no limit on the number of indicators a service user could select, there were several participants (N=10) who chose over five, with 13 indicators being the maximum chosen.

**Table 3:** Frequency of indicators chosen within each domain.

Domain	Count of indicators selected	Frequency (%)
Emotional interpersonal	9	4.6%
Emotional intrapersonal	119	60.7%
Informational interpersonal	7	3.6%
Informational intrapersonal	61	31.1%
Total	196	100%

*Total (N=196) represents 89 distinct service users, choosing on average, 2.3 indicators each*.

**Figure 3:**
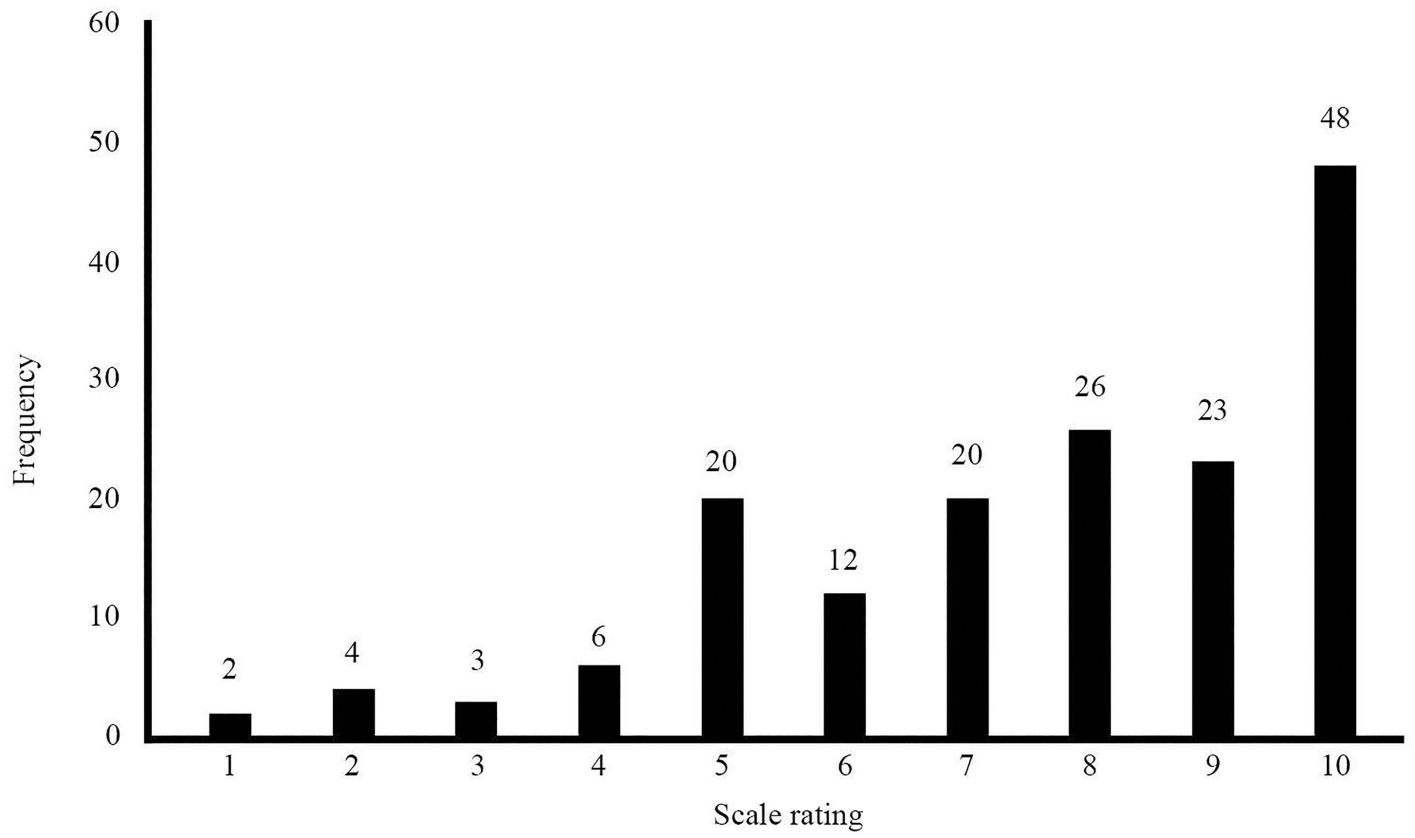
The distribution of response scores to the post-session SWAN-OM on the scale 1–10, where 1=strongly disagree to 10=strongly agree.

The results of the follow-up ratings show early indications of young people finding the measure helpful in achieving their single-session wants and needs. Figure 3 chart shows the frequency of response scores for those who completed the entire measure (N=164) which indicated if their chat session achieved what they set out to achieve (initially measured in the pre-chat indicators). Only 9% of trialled participants said that their in-session wants and needs were not met (rating<5), whereas 32% said they were somewhat met (rating between 5 and 7) and 59% responded that their session did help them achieve their session wants and needs (rating of 8–10).

##### Workshop Results: Practitioner and Young People Feedback

Overall the feedback was positive, with both practitioners and young people commenting that the SWAN-OM helped focus the chat and provided a framework for the conversation that easily allows for exploration of a topic in session. Other positive feedback from young people was that the questions were informative and interesting, it helped them generalise their thoughts and feelings into achievable wants and goals, and could be used to quickly build trust with their practitioners.

One of the most interesting, and pressing, findings of the pilot was the disparity between domain selection, with less than 10% of service users selecting indicators in the interpersonal domains. Upon reflecting on these results with the practitioners in the workshops, it became evident that this might not be a true reflection of the needs for those seeking brief support in the service, for instance, many young people come to chat to discuss problems with their relationships despite choosing an intrapersonal domain indicator. One of the reasons why this might not be reflected in the results is because of the wording of the initial interpersonal questions: ‘I want to learn some skills to try with other people’ (informational-interpersonal) and ‘I want to explore more about how I relate to other people’ (emotional-interpersonal). Some of these initial findings were used to construct the activities for the usability testing phase with children and young people in phase 4.

### Phase IV: Usability Testing

To sustain the digital use of the measure, it has to be suitable from a user-experience (UX) perspective but also be digitally feasible (i.e.: presentable on different digital screens). Workshops with young people (focus groups and surveys) were conducted to explore the usability of the measure and its design. Workshops and usability testing exercises were done iteratively with different groups of young people, refining the instrument and goals for each workshop. Formal and more informal systematic methods of data collection are used during this phase, serving different purposes and formative goals for the creation of SWAN-OM in a web-based environment.

#### Remote Workshops and Surveys With Young People

##### Participants

For the workshops, young people were recruited from external mental health advisory and patient representative groups who had experience using mental health services. All participants were presented with an information sheet and gave informed consent. Each group had between 2 and 8 participants (*n*=38, female=31; male=7) ranging from 14 to 24 years old (representative of Kooth’s service users). Sessions were virtual but not video recorded, but field notes were taken in all workshops for transcription. Workshops lasted on average 50min and all participants received reasonable monetary reimbursement of £15 for their participation.

The acceptability survey was administered to children and young people recruited from Kooth (N=8) of which 50% were female (N=4), 25% were male (N=2), 12.5% were agender (N=1) or preferred not to say (N=1) with an average age of 16 (Range=14–17, SD=0.99). Young people came from varied backgrounds with five from a white British background, two from a mixed background and one from an Indian background. Both ethnicity and gender were self-reported variables in the survey.

##### Design and Procedure

Nine workshops were conducted in total. These were all remotely done over Zoom (see footnote 1) using Miro, an interactive board software^2^ to present and guide the activities with the participants. The main aims of the workshops were to address outstanding questions, such as: how to categorise the reactive measure statements (domains vs. themes – see open and close card sort activity below), how to reduce the number of statements, whether the wording was clear and understandable, and how to best display the flow of the measure on Kooth as a platform. Open Card Sorting Workshops. Three of the nine workshops focused on open-sort card activities with young people (N=13). Card sorting is a user-centred design methodology that helps to discover how users find information in a system (Fincher and Tenenberg, 2005), open card sorting presents no constraints on the activity and allows participants to group the statements freely (Righi et al., 2013).

Participants were given a set of cards representing the initial piloted 34-item statements from the measure, participants were then asked to group and sort the cards in an appropriate way. The final groupings in each workshop were formed through consensus between participants, facilitators delivered instructions that summarised main findings from the workshop and enabled reflection on the points that summarised the workshop findings and a final round of instructions solved conflict to reach agreements between participants discrepancies. Each grouping from the workshop findings was then described by the participants to ascertain meaning that was noted in the boards of each workshop, voting was not needed as participants tended to converge into agreements within workshops. We used the workshop field notes and observational findings to reduce the number of statements and change the wording of those that were highlighted as problematic or difficult to understand during the activities.

Workshops were also used to explore the appropriateness of the four higher-order domains (1. Emotional; 2. Informational; 3. Interpersonal; and 4. Intrapersonal). After these workshops with young people and the earlier workshops with experts, six themes were formed in which the statements were categorised as: (1) ‘To understand the help I can get’; (2) ‘To share my story with someone’; (3) ‘To set and achieve goals’; (4) ‘To explore my emotions’; (5) ‘To improve my relationships’; and (6) ‘To learn ways to cope’. These themes were later one used on closed card workshops to determine the items that best fitted the themes.

Closed Card Sorting Workshops. Two of the nine workshops (*n*=10) used closed card sort activities. Closed card sorting activities constrain the users to enable constructs or categories to be tested (Righi et al., 2013). Therefore, in these workshops, six categorical themes which were predefined from the open card sorting and expert workshops were presented to participants: (1) ‘To understand the help I can get’; (2) ‘To share my story with someone’; (3) ‘To set and achieve goals’; (4) ‘To explore my emotions’; (5) ‘To improve my relationships’; and (6) ‘To learn ways to cope’. Participants were given a set of cards representing the initial piloted 34-item statements from the measure and asked to group them into the six categorical themes. Similarly, to in the open card sorting workshops, for the closed card sorting, the item allocation to themes in each workshop was formed through agreements between participants, item allocations were summarised at the session and facilitators enabled reflection on the main findings and solved conflict to reach consensus facilitators used robin rounds to all participants to encourage participation on the main points summarised from the workshops and note each participant opinion.

From the closed card sorting workshops, we found that participants were able to differentiate between intrapersonal and interpersonal wants and needs from the domains of the instrument. Participants addressed the themes as more accessible and understandable than the domains. The themes were perceived as appropriate by participants, one participant quote from one of the closed card workshops reflected this: ‘I quite like them [themes], I think it cover most of the options, and the write up option, makes me feel that Kooth is trying to get the best of the young people’, participants discussed the appropriateness of the themes for covering universal needs and wants from people accessing mental health services for the first time, it also provided with further evidence on the importance of individual personalisation of outcomes for single-sessions. Acceptability Survey. The aim of the survey was to complement the evidence and iterative changes to SWAN-OM from the open and closed card sorting activities. The survey was used to provide clarity around the acceptability of the statement wording and receive qualitative feedback about each of the final statements. A Likert scale 0–10 (0: Bad wording; 5: Nor bad nor good; and 10: Good wording) presenting each of the reduced statements (20 remaining items) was used to explore any problems with the final wording of statements. The survey also asked young people to explain their understanding of their statement in an open-ended question (‘Tell us your opinion about the statement’) and a multiple-choice question presenting the ‘themes’ to select under what category they think the statement fitted best.

##### Qualitative Analysis of the Workshops

Each workshop’s field notes were transcribed and analysed using a deductive framework analysis approach (Ritchie and Spencer, 1994; Ritchie and Lewis 2003); qualitative exploration of each collected field notes (N=25) was used, this qualitative method allowed enough flexibility to analyse this type of data. A matrix was developed establishing deductive ‘themes’ that were of interest for the study: (a) Domains as an intuitive way to understand support for young people and Establishing new themes (or ways) to group statements; (b) Suggestions for new statements; Reduction of statements; and Comments about wording.

The content of the field notes was coded and analysed by two Kooth researchers with no previous relationships with participants. Researchers familiarised themselves with raw data and provided the framework of exploration for the *a priori* defined questions of interest about the measure (Srivastava and Thomson, 2009). Extracts from the field notes were identified, coded and indexed within the framework, data from each workshop were charted to present the information in the matrix and summaries of all workshops were created for each theme. The findings were presented to the workshop facilitators to ensure the interpretations were grounded in the experiences of participants.

##### Results

Quadrant and Theme Exploration. Participants unanimously agreed that categorising the statements by ‘themes’ was perceived as less difficult than categorising by the domains. For example, comments from participants like ‘they are all [statements] about accepting your emotions, exploring feelings and being ok’ provided information to create a ‘theme’ named ‘Exploring my emotions’, while other comments, such as: ‘these statements are about finding out how to get support on Kooth and staying in control’ exemplify how the theme ‘To understand what help I can get’ was created, other themes like ‘To learn new ways to cope’ arose from the work with young people in the workshops ‘They’re all [grouped statements] about identifying a problem and managing it: how to fix things and get what you want. [Participant E] suggested the term “coping with your problems”’, coping seemed to resonate with participants in different workshops and be part of the lexicon of young people when looking at statements from the measure one participant said, ‘Maybe you can group something together about coping mechanisms or self-care’.

This provided enough evidence to discard the quadrants as a way to display and organise the statements and favour the use of ‘themes’ in the measure as an intuitive way to display and group the statements. These themes were taken into the closed card sorting exercises to be tested.

Reduction of Items and Changes to Item Wording. The original 34-items produced for the measure were identified by product developers and designers as too overwhelming and lengthy for a digital environment. Observations in the open and closed card sorting workshops were captured surrounding relevance, confusion, ambiguous meaning or similarities between items perceived by participants. Some items were identified as being too similar to each other, making the case to reduce the total number of statements by combining some. This was reflected in comments, such as ‘To identify solutions to manage my relationships’ and ‘To identify goals that will help me improve my relationships’, which are very similar. This led to the development of a new item ‘To identify solutions to improve my relationships’. When tested, some items were misread or confused, these items were therefore clarified. Some items were about ambiguous concepts, for instance, ‘safe space’ was perceived as very subjective by young people: ‘safe space is a bit vague, it has many possibilities’. Therefore, the original statement ‘A safe space to explore how I feel’ was revised into ‘to explore how I feel’.

As a result of the card sorting workshops, a final number of 20-items were kept or amended out of the original 34-items. Acceptability Survey Results. Overall young people understood each of the statements (Table 4), with the average level of understanding of all statements being ranked at 7.54 (SD=0.53). These results help to provide further evidence on the changes that the measure experience after the iterative changes of usability testing.

**Table 4:** Average score for each item’s survey acceptability.

Statement	Average	SD
To feel safe in my relationships	7.25	1.11
To be able to open up to people in my life	8.50	0.95
To be comfortable asking for help outside Kooth	7.88	1.77
To explore difficulties in my relationships	7.38	1.40
To talk about something personal to me	7.00	2.14
To feel better	7.75	2.21
To explore how I feel	7.38	1.62
To feel listened to	7.63	1.99
To be more comfortable with my feelings	7.00	1.41
To learn how to relate to other people	6.88	1.63
To learn how to manage conflict with others	7.75	1.57
To identify solutions to improve my relationships”	6.75	2.82
To find out how helpful it is to talk to someone	6.50	2.41
To learn how to feel better	7.88	1.35
To identify a solution to a problem in my life	7.75	1.90
To learn the steps to achieve something I want	7.13	1.83
To identify ways to help me worry less	7.88	1.35
To find ways I can help myself	7.25	1.38
To find information about how to keep myself safe	8.38	1.50

*The average represents the mean score on a scale of 1 to 10, with SD representing the Standard Deviation*.

The open-ended question responses were assessed for each statement to determine whether the overall opinion for each statement was positive, negative or neutral. A Fleiss kappa was computed to assess the agreement between three raters in the perception of the statements. Fleiss’ Kappa showed that there was a fair agreement between the three randomly selected unique rater’s scores, K=0.23, *p*<0.001. Thus, we can conclude that the young people had moderate positive opinions on the item statements.

Participants generally agreed that the measure would be acceptable and useful to young people. For example, one participant said ‘I like that it’s down to the point though and tells you in simple terms what practitioners want’; another commented that it ‘definitely was not overwhelming’; and another said they thought it was ‘really good because it might help to organise your feelings’. Some of this feedback collected in the survey open-ended questions synthesise the goals and aims for the SWAN-OM as an I-PROM measure of single-session that is adequate for young people accessing a web-based mental health service.

## Discussion

In this paper, we discuss a novel method to design an outcome measure within a digital therapeutic setting for single-session therapeutic support. With this research, we aim to highlight the strengths of designing outcome measures with a digital context in mind and the value of engaging in iterative design from item generation and reduction to usability testing and participant engagement. Here, we discuss the key elements of the design process as well as our lessons learnt and limitations and strengths of this design.

Single-sessions and brief interventions are being increasingly used to provide therapeutic support and have been seen to be an effective method of therapeutic support (Schleider and Weisz, 2017). However, there are limited ways to measure the impact or benefit of these interventions, especially at scale. Importantly, a fully nomothetic or clinical symptomatic-based outcome measure is not suitable for this type of therapeutic intervention, as discussed in the introduction to this paper, we would not expect a change in symptomatology directly after one therapy session. I-PROMs, however, are useful as they align more with patient-perceived progress rather than measurement being centred around the clinician perceived symptomatic progress (Collins, 2019; Flannery and Jacob, 2020) but still allow for aggregation of scores. Therefore, we identified that this style of measures is particularly appropriate for measuring outcomes from single-session or drop-ins as they are commonly informative, asset-based and solution-focussed (Dryden, 2020; Kachor and Brothwell, 2020). It is therefore critical to align a novel outcome measure being designed to the type of intervention to appropriately assess and measure outcomes. Consequently, in this paper, we described the method of designing a new, digitally enabled I-PROM for single-session therapeutic interventions that directly measures service user wants and needs as they enter their therapeutic chat session.

Developing an instrument with pre-session item selection and post-session measurement of wants and needs for single-session, provided the most suitable method of capturing outcomes from the intervention. The novel combination providing common wants and needs alongside a free-text option enables the alignment with the expected outcomes from this type of person-centred therapeutic intervention. The instrument provides the flexibility for both users who come with individualised specific needs, as well as those who need guidance to structure their needs and wants for a single-session. Specific considerations were taken around the wider implications of designing an effective instrument for young people in the age of digital technology and the move to web-based therapeutic chat sessions, such as the sessions delivered at Kooth. The key stages of developing this digital person-centred measure were item creation, content, face validity and usability testing. We took an iterative design process to enable small but substantial changes to the measure to be made to ensure usability, clarity and accessibility of the measure (Mohr et al., 2017) for young people in a digital web-based therapy service. The ongoing aim is that the SWAN-OM can be used not only in the tested service, but also in wider web-based and face-to-face services that utilise computer-based outcome measures but that are importantly aligned with person-centred therapeutic support and offer single-sessions.

As the SWAN-OM has been designed in the long term for a range of services, it was important in the design process that experts, practitioners and service users were involved in the development of the measure, both in relation to the items and measurable constructs, but also the experience of using the measure and its therapeutic application; hence, we put users and main stakeholders at the centre of the design process (Honary et al., 2018). This led to an agile way to design particularly suited to the development of measures for a digital or technology-assisted therapeutic service, combining classical methods of survey development and novel methods of participatory research to achieve an optimal user experience.

Overall the current feedback from both practitioners and young people throughout the design process provided positive comments about the SWAN-OM, saying that it helped focus the chat and provided a framework for the conversation that easily allows for exploration of a topic in a single or drop-in session. Other positive feedback from children and young people was that the questions were informative and interesting, it helped them generalise their thoughts and feelings into achievable goals and could be used to quickly build trust with their practitioners.

From the current pilot and usability testing, we obtained positive feedback and rich qualitative evidence for the feasibility and accessibility of the measure. However, when using the SWAN-OM there could be an effect of social desirability on SWAN-OM outcomes. As young people provide agreement ratings of how much they achieved what they wanted from their session with a practitioner, there may be a social pressure to positively rate their outcomes. In the digital therapeutic space, this may be less challenging to overcome than in a face-to-face therapeutic environment where the young person is identifiable and in a room with a practitioner. There is, however, some evidence to suggest that on wellbeing scales, social desirability bias only has a modest effect (Caputo, 2017). Yet it is still a consideration to take forward into future validation studies and iterations of the SWAN-OM design.

We have established some lessons learnt that illustrate the benefits and challenges of designing a novel outcome measure in a digital environment. In the item, generation phase where we developed a set of statements that are responded to before the chat which represents the wants and the needs for young people an understanding of the therapeutic outcomes of single-sessions was crucial. Previous work conducted with Kooth on their Theory of Change (Hanley et al., 2021) was instrumental in determining expected wants, need and outcomes of single-sessions (Hanley et al., 2021). This then enabled an initial item generation which mapped into Kooth’s Theory of Change quadrants as main domains or constructs of the measure. Starting from a wider set of items enabled a systematic reduction of item statements through using content validity indexes and engaging with experts. This allowed us to evaluate the quality and relevance of items at an early stage. We found that researchers and designers may find it difficult to otherwise engage with the reduction of the item statements. Use systematic methods for reduction by using content validity indexes, and early engagement with an ERG may help to reduce bias and accelerate the design and development process of the instrument.

It is important to test the reduced number of items for face validity with a pilot within the web-based therapy service Kooth. From doing this, we were able to evaluate within the design process the acceptability of the SWAN-OM to service users and practitioners. Piloting the instrument in the relevant context is of key importance to get initial findings on the instrument scores and scales, as well as to examine outliers in answering the measure. In a digital context, software engineering and product developer’s perspective need to be considered, how the measure will look from a UX perspective and the technical requirements that the instrument will need to be successfully implemented in the service. For the SWAN-OM, regular workshops while piloting involving the software engineering teams and developers enabled us to capture their views and technical preferences while inform them of the practitioners’ feedback and research findings that contribute to the next phase of measure development.

The usability phase requires direct engagement with the population of interest, remote workshops can be a useful tool to overcome some barriers of access to the population that may be difficult to reach due to locations or time constraints. We recommend engaging experts by experience, as they can provide further insights into the frustrations and difficulties that service users face in their usual care. Different data collection tools can be used within the workshops and they may serve to gain insights on the design and wording of the tool and its statements, it is important to design workshops that are engaging for participants, and be open to change activities and purpose as research questions on testing may change as findings develop from each workshop iteratively. This type of engagement, though highly beneficial to agile outcome measure development, does lead to constraints in measure evaluation and this is something that needs weighing up throughout the process. As this measure development was conducted in an applied setting the iterative analysis of qualitative findings may influence some bias in the findings, but we hope this paper has demonstrated the benefit of this phased approach to develop an instrument and how to overcome some of the challenges that the researcher will find in similar contexts when developing an outcome measure aiming to demonstrate validity.

An important link to the type of therapeutic sessions the SWAN-OM provides measurement for is that they are single-sessions or drop-in session in which is assumed the service user may not come back for follow-up. Therefore, as the SWAN-OM is intended to act as an I-PROM, the instrument provides a mechanism for the service users to focus and reflect on what they would like to achieve in that session without having to respond to all items, in addition, to provide choice by selecting a ‘want’ or ‘need’ of their own if required. This makes the measure quick to complete and very tailorable to individual needs but may compromise the structural validity of the instrument. Articulating wants and needs relating to mental health and emotions is difficult especially for children and young people who may not have the mental health literacy or experience using emotionally descriptive language (Burns and Rapee, 2006) to self-direct these important conversations in chat. Consequently, design decisions with the SWAN-OM development resulted in providing six initial themes that allowed the service user to narrow down what they want to focus the chat on and then select up to three specific items within the selected themes, those items are representative of common wants and needs often seen in the service population. This decision to have a two-phased pre-session measurement, by providing initial themes and sub-theme items allowed a more natural display of the statements in a digital environment. This is intended to reduce the cognitive load on the service user (Sweller, 2011) and importantly avoid potential distress when presenting all statements at once. This design decision also allowed the measure to meet digital platform accessibility standards (WCAG 2.1, 2018).

There are drawbacks to the SWAN-OM being a two-phased, logic-dependent measure as not all items will be presented to all service users. Items are presented based on the themes chosen by the service users. There is no limit on the number of themes they can select but there is a limit on the number of item statements service users can select. This structural design makes it difficult to test psychometric properties of the SWAN-OM in a traditional way, such as factorial analysis, but concurrent and structural validity will still be examined in future studies relating to the ongoing development of the SWAN-OM and its novel structure as an I-PROM. The structural design of outcome measures needs to be considered especially in digital environments with flexibility around how the measure is presented. We recommend thinking about the knock-on effects of these decisions should be considered in advance, especially when designing later validation studies to determine what psychometric properties should be prioritised during the design and development process of the instrument, like the structure or dimensions of the instrument can be compromised.

Directly related to the design decisions and critical to the development of the SWAN-OM was the participatory approach. Involving children and young people in the conceptual and physical design and involving experts and practitioners from inception. This participatory approach ensured that the measure was usable, desirable and feasible to be implemented in a digital web-based therapy service. For example, by working with the practitioners from Kooth, we narrowed down the numbers of items service users can select to three items. This number was chosen to ensure that the young people’s expectations of the session were managed and that their wants and needs were attainable as service users may have wanted no limitation in the selection of statements as their chat expectations to differ from practitioners and their capacity to meet those wants and needs. Young people also suggested adding a free-text option where children and young people can write their own bespoke ‘want’ or ‘need’ which their practitioner will get to see before the session. This importantly provides a space for service users to write bespoke session wants or needs that may not fit into the themes or statement options that SWAN-OM provides, even though these are relatively broad by design this ensures a person-centred approach to measure and maintains the idiographic nature of the instrument. An interesting and vital study will be needed to explore the wants and needs of the service users free-text responses. It will be useful to explore whether free-text responses align with any of the items commonly presented in the instrument and if there are underlying repeating themes there that might suggest other items needing to be added to the SWAN-OM or further domains are yet to be discovered representing the wants or needs of users accessing a web-based therapy service in a single-session fashion. Additional analysis and exploration into the use of the measure may inform and modify the SWAN-OM further and this demonstrates the value of reflection and iterative design in developing outcome measures.

In the development of outcome measures, in particular digital measures where the service users and practitioners are not in the same physical space, there is increased importance on using participatory involvement in the design to test the appropriateness of items and increase engagement with the measure before this is used more widely in therapeutic sessions. On the whole, the young people in the participatory workshops felt that the items were broad enough but still dealt with common issues facing young people; for example, we received comments from the workshops that the statement ‘To learn how to feel better’ was said to be ‘A broad statement that directs the person towards coping techniques and advice’ and in response to the statement ‘To feel listened to’ a young person commented that ‘this is an excellent statement and very clearly outlines a problem that many young people face today where they aren’t listened to and can easily let people give specific advice to the young person’. The young people who provided feedback in the workshops around the pre-session theme selection stipulated that this made the process less overwhelming for the children and young people to use and determine what they wanted or needed from a therapeutic chat session. Young people provided valuable feedback on the item statements as well; for example, ‘To explore how I feel’ rendered positive comments from participants, such as: ‘This is a useful topic, and the statement is direct, while still covering many topics’. Additionally, young people stated that when service users select a statement, such as this ‘they want to learn more about how they feel’ is a useful item as ‘sometimes it’s hard for yourself to know that’, and this statement allows them to therefore express this to the practitioner. This feedback is encouraging and combined with the pilot trial data from the face validity phase suggest the SWAN-OM measure is usable and acceptable for single-session therapeutic interventions from the viewpoint of the young people, who represent the age group of the service users who would use this instrument. Despite some participants of the workshop were neurodivergent, further research specifically targeting neurodivergent groups, such as dyslexia, autism spectrum and other conditions, may bring further insights on the interpretation of wants and needs displayed in the instrument, individual differences within these populations may influence the variability of scores and influence understanding and interpretation of the statement reducing effeteness. Working with neurodivergent populations may address other accessibility issues, some of them were raised in the workshops by participants, such as not having time pressure to complete the instrument, having the option to skip and to personalise the statements to tailor the session to the individual.

Using an iterative design process was beneficial to the development of the digital measure. However, it is difficult to work across practitioners, young people and software engineers who are building the measure. There can be some competing needs from these stakeholder groups, the software engineers and product managers may have specific needs for the measure to be accessible on a range of digital devices, whereas practitioners want something that aligns with their practice and service users are seeking a measure that helps them and can feel identified with while being easy and quick to use. This led to changes in the SWAN-OM, for example, there was a change from a 10-point scale to a 5-point scale to increase the compatibility on digital devices. Interestingly, some changes like this converged with feedback from the young people, here, they reported that the 10-point scale was too overwhelming and a 5-point scale was more intuitive. This is in line with prior literature, particularly into how younger children engage with Likert scales (Chambers and Johnston, 2002). Primarily children have been seen to engage in Likert scales with 3 and 5 points very similarly (Chambers and Johnston, 2002). Other research also shows similar responses across different scale lengths (Taherdoost, 2019). It is worth noting that using scales with 7–10 point ranges produces more reliable responses and more information from the respondents (Taherdoost, 2019). Nevertheless, it is important to balance scale reliability and response criterion validity with scale accessibility with the children and young adult audience in mind. This is particularly relevant as the younger service users engaging with the measure will be only 11years old. It was, therefore, important to listen to the young people’s feedback which is consistent with this, and given the information on the lack of change in responses due to scale length, we decided this was a valid change to make to the measure.

There was also converging feedback from service users and product developers for the change from quadrants to six themes. This highlights the need to be responsive in outcome measure design as even if the theoretical structure maps nicely to quadrants, if this is not intuitive or understood by the service users and only two quadrants are being selected from a product and measurement perspective this is not an effective tool. Ergo, even though there are varying needs from these participatory groups, the outcomes from the steering and feedback provide a more accessible and user-centred measure that aligns as an I-PROM. The iterative design process used allowed for changes like this to the instrument, without SWAN-OM being re-designed or halted in progress, the use of systematic methods for questionnaire development from psychometric literature (e.g. I-CVI) was also helpful to continue validating the changes made since foundation (Rattray and Jones, 2007).

Yet as discussed there are several limitations to be considered and this paper importantly represents the early development of a measure for the specific context of Kooth, which is the digital platform in which the measure was designed with the service in mind. This limits the generalisation of our findings beyond the relevance of this context and potentially the measure. Despite digital mental health services growing, transferring this tool and findings to other digital services or face-to-face services presents challenges that are beyond the scope of this paper. Of importance to note here, the nature of the constructs and domains and the purpose of the tool are yet to be examined in more detail and investigate the relevance of SWAN-OM measurement to person-centred outcomes and preventative interventions, and if those are aligned with the solution-focused approaches for single-session and drop-in interventions that take place at Kooth. Goals and idiographic measures can also be difficult to interpret and aggregate as a proxy of mental health improvement or goal achievement, as well as determine the thresholds for meaningful change statistically.

The next steps are to validate the SWAN-OM by comparing them to other measures, such as the Positive and Negative Affect Scale (Watson et al., 1988) to explore the immediate short-term emotional changes as a result of the intervention, the Experience of Service Questionnaire (Brown et al., 2014) to explore the experience and satisfaction with care and the YCIS (Riemer and Kearns, 2010) to evaluate the quality of the processes and intervention. These instruments will aim to test the concurrent validity of the SWAN-OM following the Donabedian framework (Donabedian, 1988) for quality of care. For this, the SWAN-OM will be implemented at Kooth Children and Young people’s service as a routinely collected outcome measure for drop-in and single-sessions within the service. This will allow us to understand if the SWAN-OM outcome scores align with changes in emotional states before and after a chat session alongside with young people’s perception of session satisfaction and positive impact. This future research will also provide further evidence about the instrument validity and may spur reduction of some statements that are not frequently selected within the service, transforming the measure. Moreover, future research should be exploring the transaction from a digital context to a physical context to find out the usability of the SWAN-OM across mental health services as a valid outcome measure for single and drop-in sessions for young people beyond the web-based therapy service Kooth.

In conclusion, the SWAN-OM development and design process demonstrate the complexities of designing an idiographic outcome measure for (1) digital settings and (2) for single therapeutic or drop-in sessions. In this research, we demonstrate a route to measure creation that is both integrated into a digital platform but also aims to apply to other digitally enabled settings, such as face-to-face therapeutic services, that offer single-session or drop-in services. The phased approach demonstrates the value of using theory and research literature to drive item generation and content validity of an outcome measure but also highlights the importance of the participatory research that involves stakeholders, such as experts, practitioners and more importantly young people, in shaping the design of the instrument and improve its face validity and usability as a patient-reported outcome that is user-centred and person-centre. This development process that is iterative and responsive to feedback and needs of the stakeholders from whom the measure is used causes limitations for a systematic design process and research methodologies and may add complexities for further validation stages to demonstrate good psychometric properties. Nevertheless, this phased approach enabled a more usable and appropriate outcome measurement for the targeted intervention, where none of the previously used instruments in the literature aligned with. We hope SWAN-OM will pave the way for accelerated digital outcome measure creation, filling the gap to measure single-session and drop-in interventions in children and young people. But also to motivate fellow researchers to embark on participatory approaches to the design, validate and identify appropriate outcomes measures that demonstrate the value and therapeutic potential of single-session and drop-in therapeutic contacts in the children and young people population.

## Data Availability Statement

The raw datasets presented in this article are not readily available because they contain information that can compromise the privacy of the research participants. Requests to access the datasets that support the findings of this study should be directed to research@kooth.com, upon reasonable request.

## Ethics Statement

All procedures followed were in accordance with the ethical standards of the responsible committee on human experimentation (institutional and national) and with the Helsinki Declaration of 1964 and its later amendments. Ethical review and approval were not required for the study on human participants in accordance with the local legislation and institutional requirements for service quality improvement. Written informed consent to participate in this study was provided by the participants’ legal guardian/next of kin.

## Author Contributions

SG and AS contributed to the conception and design of the study. LS and SG co-first authors organised the database, performed the analysis, wrote the first draft, and contributed equally to the manuscript. TH and AS wrote sections of the manuscript. All authors contributed to manuscript revision, read, and approved the submitted version.

## Funding

This work has been funded as part of service innovation by Kooth plc. There has been no external funding sought to carry out this work. As the funder of the project Kooth plc. provided the resource to plan for, collect and analyse data, and write the report for this study.

## Conflict of Interest

SG, LS, and AS are researchers employed and receive honorarium by Kooth plc.

The remaining author declares that the research was conducted in the absence of any commercial or financial relationships that could be construed as a potential conflict of interest.

## Publisher’s Note

All claims expressed in this article are solely those of the authors and do not necessarily represent those of their affiliated organizations, or those of the publisher, the editors and the reviewers. Any product that may be evaluated in this article, or claim that may be made by its manufacturer, is not guaranteed or endorsed by the publisher.

## Footnotes

^1^ https://zoom.us/

^2^ https://miro.com/
