# Supplementary material for: The Session Wants and Need Outcome Measure: The Development of a Brief Outcome Measure for Single-Sessions of Web-Based Support
Source: Front Psychol. 2021 Oct 29;12:748145. doi: 10.3389/fpsyg.2021.748145 (PMC8588807; doi:10.3389/fpsyg.2021.748145)
Supplement: Supplementary file 1 [file Table_1.DOCX]

Supplementary Material

# Supplementary Tables

Supplementary Table 1: *Initial pool of items for reactive measure*

| **Domain** | **Theme** | **Item** | **Statement pre-chat** | **Statement post-chat** |
| --- | --- | --- | --- | --- |
| Emotional Intrapersonal | Seeking Safety | Item 1 | To feel safe to tell others in my life what is going on | I now feel safer to tell others what is going on |
|  | Expressing Relationship Breakdown | Item 2 | Relationships around me are difficult | I now know how to manage difficult relationships around me |
|  | Opening Up - Disclosure | Item 3 | To share my my story in a way that has not been possible before | I was able to share my story |
|  | (difficult to open up to local support) | Item 4 | To feel comfortable opening up to those around me | I now feel more comfortable about opening up to others |
|  | (difficult to open up to local support) | Item 5 | To feel comfortable accessing support offline | I now feel more comfortable asking for support offline |
|  | Exploring curiosities | Item 6 | To explore what is possible in my relationships | I understand what is possible in my relationships |
|  | Finding a solution | Item 7 | To resolve some issues in my relationships | I have been able to resolve some issues in my relationships |
|  | Seeking clarity | Item 8 | To be able to understand others | I was able to ask questions to help me to understand others |
|  | Exploring feelings | Item 9 | To understand better how others might feel about things | I understand better how others might feel about things |
|  | Able to show gratitude | Item 10 | To receive help from someone | I feel grateful for the help I received |
|  | Able to offer feedback on the impact of the session | Item 11 | To share my story and feel listened to | I appreciated that someone listened to my story |
|  | Offloading key concerns | Item 12 | To explore my problems with someone | I was able to tell someone about my problems |
| Emotional intrapersonal | Seeking Safety | Item 13 | To feel in control of what I share | I did feel in control of what I shared |
|  | Seeking Safety | Item 14 | A safe space to explore how I feel | I got a safe space to explore how I feel |
|  | Requiring immediate emotional relief | Item 15 | To feel better now | I feel better |
|  | Requiring immediate emotional relief | Item 16 | To explore all my options | I have been able to explore my options |
|  | Identifying Inner resources/Coping mechanisms | Item 17 | To discover how I can help myself to feel better | I can now help myself feel better |
|  | Expressing Relationship Breakdown | Item 18 | I don't have anyone to turn to | I now feel like I could turn to someone |
|  | Opening Up - Disclosure | Item 19 | To talk about something I haven't told anyone before | I was able to talk about something I haven't told anyone before |
|  | Exploring curiosities | Item 20 | To explore what is possible in my life | I was able to explore what is possible in my life |
|  | Exploring curiosities | Item 21 | To explore what is possible on Kooth | I understand what is possible on Kooth |
|  | Offloading key concerns | Item 22 | Take the space to get things off my chest | I was able to get things off my chest |
|  | Achieving self efficacy | Item 23 | To be able to work out a situation I am in | I was able to work out the situation I was in |
|  | Validating feelings | Item 24 | To feel listened to | I felt listened to |
|  | Validating feelings | Item 25 | To be ok with my feelings | I am ok with my feelings |
|  | Exploring feelings | Item 26 | To explore how I feel | I was able to open up about my feelings |
|  | Taking control of support | Item 27 | To feel in control of how I receive my support | I had a say in what we talked about |
|  | Seeking clarity | Item 28 | To understand my feelings and behaviours | I understand my feelings and behaviours better |
| Informational interpersonal | Identifying coping Mechanisms / skills | Item 29 | Discover how to find the people who can help me | I can now identify people who might be able to help me |
|  | Exploring coping mechanisms | Item 30 | To learn how to relate with others | I can identify new ways to relate to others |
|  | Tangible goals/outcomes | Item 31 | To identify goals that will help me improve my relationships | I know the steps to take to improve my relationships |
|  | Expressions of relationship breakdown | Item 32 | To understand or improve my relationships with others | I have the tools to better understand my relationships with others |
|  | Expressions of relationship breakdown | Item 33 | To learn how to manage conflict with others | I now feel more confident managing conflict with others |
|  | Solution focused | Item 34 | To solve my problems with others | I was able to find solutions to my problems with others |
|  | Able to offer feedback on the impact of the session | Item 35 | To find out how useful it is to talk to someone | I found it useful talking to someone |
| Informational intrapersonal | Seeking safety | Item 36 | Some information about how to keep myself safe | I got some information about how to keep myself safe |
|  | Requiring immediate emotional relief | Item 37 | To find ways to help me worry less | I have found some ways to help me worry less |
|  | Identifying coping mechanisms / skills | Item 38 | To learn how to feel better | I have learned ways/skills to feel better |
|  | Identifying coping mechanisms / skills | Item 39 | Able to manage my situation better | I feel able to manage my situation better |
|  | Identifying inner resources | Item 40 | To identify ways I can help myself | I have identified ways to help myself |
|  | Tangible goals/outcomes | Item 41 | To learn the steps to achieve something I want | I understand the steps to achieve my goal |
|  | Tangible goals/outcomes | Item 42 | To make progress on tackling a specific problem in my life | I now know the steps to take to address a specific problem |
|  | Expressing relationship breakdown | Item 43 | To understand relationships better | I now understand my relationships better |
|  | Achieving self efficacy | Item 44 | Information on how to feel more confident | I feel more confident in my abilities |
|  | Finding a solution | Item 45 | To explore solutions to my problem | I have found a solution to my problem |
|  | Seeking clarity | Item 46 | To be clear on what the main issue is in my life | I now feel clear about the main issue in my life |
