# Supplementary material for: The Session Wants and Need Outcome Measure: The Development of a Brief Outcome Measure for Single-Sessions of Web-Based Support
Source: Front Psychol. 2021 Oct 29;12:748145. doi: 10.3389/fpsyg.2021.748145 (PMC8588807; doi:10.3389/fpsyg.2021.748145)
Supplement: Supplementary file 2 [file Table_2.DOCX]

Supplementary Material

# Supplementary Tables

Supplementary Table 2: *Initial 34-Item Content Validity Index and Kappa*

| **Item** | **I-CVI Relevance** | **Pc** | **Kappa** | **I-CVI**  **Clarity** | **Pc** | **Kappa** |
| --- | --- | --- | --- | --- | --- | --- |
| **Emotional Interpersonal domain** | | | | | | |
| Item 1 | 0.875 | 0.004464286 | 0.874439462 | 0.75 | 0.010416 | 0.7473684 |
| Item 2* | 0.625 | 0.01875 | 0.617834395 | 0.75 | 0.010416667 | 0.747368421 |
| Item 3 | 0.625 | 0.01875 | 0.617834395 | 0.875 | 0.004464286 | 0.874439462 |
| Item 4 | 1 | 0 | 1 | 1 | 0 | 1 |
| Item 5* | 0.75 | 0.010416667 | 0.7473684 | 0.375 | 0.052083333 | 0.340659341 |
| Item 6 | 0.75 | 0.010416667 | 0.7473684 | 0.75 | 0.010416667 | 0.7473684 |
| Item 7 | 0.875 | 0.004464286 | 0.874439462 | 1 | 0 | 1 |
| **Emotional intrapersonal domain** | | | | | | |
| Item 8 | 1 | 0 | 1 | 0.875 | 0.004464286 | 0.874439462 |
| Item 9 | 0.875 | 0.004464286 | 0.874439462 | 0.75 | 0.010416667 | 0.7473684 |
| Item 10 | 1 | 0 | 1 | 0.75 | 0.010416667 | 0.7473684 |
| Item 11 | 0.875 | 0.004464286 | 0.874439462 | 0.875 | 0.004464286 | 0.874439462 |
| Item 12 | 0.875 | 0.004464286 | 0.874439462 | 0.875 | 0.004464286 | 0.874439462 |
| Item 13 | 0.75 | 0.010416667 | 0.7473684 | 0.875 | 0.004464286 | 0.874439462 |
| Item 14 | 0.75 | 0.010416667 | 0.7473684 | 0.75 | 0.010416667 | 0.7473684 |
| Item 15 | 0.875 | 0.004464286 | 0.874439462 | 0.875 | 0.004464286 | 0.874439462 |
| Item 16 | 0.875 | 0.004464286 | 0.874439462 | 1 | 0 | 1 |
| Item 17 | 0.875 | 0.004464286 | 0.874439462 | 0.75 | 0.010416667 | 0.7473684 |
| Item 18* | 0.75 | 0.010416667 | 0.7473684 | 0.5 | 0.03125 | 0.483870968 |
| **Informational interpersonal domain** | | | | | | |
| Item 20 | 0.75 | 0.010416667 | 0.7473684 | 0.875 | 0.004464286 | 0.874439462 |
| Item 21 | 0.75 | 0.010416667 | 0.7473684 | 0.75 | 0.010416667 | 0.7473684 |
| Item 22 | 0.75 | 0.010416667 | 0.7473684 | 0.875 | 0.004464286 | 0.874439462 |
| Item 23 | 0.875 | 0.004464286 | 0.874439462 | 0.875 | 0.004464286 | 0.874439462 |
| Item 24 | 1 | 0 | 1 | 1 | 0 | 1 |
| Item 25* | 0.875 | 0.004464286 | 0.874439462 | 0.625 | 0.01875 | 0.617834395 |
| Item 26* | 0.75 | 0.010416667 | 0.7473684 | 0.625 | 0.01875 | 0.617834395 |
| **Informational intrapersonal domain** | | | | | | |
| Item 27 | 0.875 | 0.004464286 | 0.874439462 | 0.875 | 0.004464286 | 0.874439462 |
| Item 28 | 0.875 | 0.004464286 | 0.874439462 | 0.75 | 0.010416667 | 0.7473684 |
| Item 29 | 1 | 0 | 1 | 1 | 0 | 1 |
| Item 30 | 1 | 0 | 1 | 0.75 | 0.010416667 | 0.7473684 |
| Item 31 | 1 | 0 | 1 | 1 | 0 | 1 |
| Item 32 | 0.875 | 0.004464286 | 0.874439462 | 0.875 | 0.004464286 | 0.874439462 |
| Item 33 | 0.75 | 0.010416667 | 0.7473684 | 0.75 | 0.010416667 | 0.7473684 |
| Item 34* | 0.75 | 0.010416667 | 0.7473684 | 0.625 | 0.01875 | 0.617834395 |

* Items included after review with the Expert Reference Group

Kappa = (I-CVI – Pc)/ (1- Pc); Pc = [N!/A!(N-A)!]* 0.5N. Pc=Probability of chance; N = number of experts; and A = number of experts that agree the item is relevant or clear.

Note: Kappa values above 0.74 are considered excellent, between 0.60 to 0.74 good and 0.40 to 0.59 fair.
